# IL-7Rα and L-selectin, but not CD103 or CD34, are required for murine peanut-induced anaphylaxis

**DOI:** 10.1186/1710-1492-8-15

**Published:** 2012-08-31

**Authors:** Steven Maltby, Erin J DeBruin, Jami Bennett, Matthew J Gold, Matthew C Tunis, Zhiqi Jian, Jean S Marshall, Kelly M McNagny

**Affiliations:** 1 The Biomedical Research Centre, University of British Columbia, Vancouver, BC, Canada; 2 Department of Microbiology & Immunology, Dalhousie University, Halifax, NS, Canada

**Keywords:** Anaphylaxis, Animal model, Food allergy, Immunity, Peanut allergy

## Abstract

**Background:**

Allergy to peanuts results in severe anaphylactic responses in affected individuals, and has dramatic effects on society and public policy. Despite the health impacts of peanut-induced anaphylaxis (PIA), relatively little is known about immune mechanisms underlying the disease. Using a mouse model of PIA, we evaluated mice with deletions in four distinct immune molecules (IL7Rα, L-selectin, CD34, CD103), for perturbed responses.

**Methods:**

PIA was induced by intragastric sensitization with peanut antigen and cholera toxin adjuvant, followed by intraperitoneal challenge with crude peanut extract (CPE). Disease outcome was assessed by monitoring body temperature, clinical symptoms, and serum histamine levels. Resistant mice were evaluated for total and antigen specific serum IgE, as well as susceptibility to passive systemic anaphylaxis.

**Results:**

PIA responses were dramatically reduced in IL7Rα^−/−^ and L-selectin^−/−^ mice, despite normal peanut-specific IgE production and susceptibility to passive systemic anaphylaxis. In contrast, CD34^−/−^ and CD103^−/−^ mice exhibited robust PIA responses, indistinguishable from wild type controls.

**Conclusions:**

Loss of L-selectin or IL7Rα function is sufficient to impair PIA, while CD34 or CD103 ablation has no effect on disease severity. More broadly, our findings suggest that future food allergy interventions should focus on disrupting sensitization to food allergens and limiting antigen-specific late-phase responses. Conversely, therapies targeting immune cell migration following antigen challenge are unlikely to have significant benefits, particularly considering the rapid kinetics of PIA.

## Introduction

Food allergies affect a significant portion of the population, with direct effects on health and quality of life. Of all food sensitivities, peanut allergies account for the most fatalities [1] and exposure to peanut antigen in affected individuals results in severe, rapid, systemic anaphylactic responses. Despite the severity of peanut anaphylactic responses, few effective treatments or therapies exist and most focus on limiting allergen exposure and management of symptoms. While peanut allergy prevalence is relatively low (estimated ~1-2% of the total population), the consequences of exposure are high and the effects of peanut allergy are disproportionately large in society [2,3].

In affected individuals, peanut-specific IgE antibodies bind to FcεR on mast cells and basophils, and are cross linked by peanut antigens, resulting in rapid release of immune mediators including histamine, leukotrienes, prostaglandins and platelet-activating factor following exposure (as reviewed in [4]). These mediators contribute to a range of pathological symptoms, including increased vascular permeability (resulting in localized edema, decreased blood pressure, and rapid decrease in body temperature), diarrhea and vomiting, and fatal respiratory failure without treatment.

To explore mechanisms underlying this pathology, a mouse model of peanut-induced anaphylaxis (PIA) was established, which closely approximates the clinical symptoms and pathology observed in peanut-allergic individuals [5]. Mice are sensitized by weekly oral feedings of peanut antigen with adjuvant. Subsequent interperitoneal challenge with peanut protein results in rapid mast cell degranulation, elevated serum histamine, and decreases in blood volume and body temperature. This model has been utilized successfully to highlight the role of B cells, CD40 ligand and mast cells and the effects of therapeutic interventions (blocking histamine and/or platelet activating factor) on peanut-induced anaphylactic responses [5,6]. In a related fatal PIA model, the importance of mast cells, macrophages, IgG and IgE have also been reported [7]. Similarly, in an adjuvant based model of PIA, treatment by a CD4 blockade could provide protection from disease, by increasing the frequency of T_reg_[8]. However, no studies have focussed on other adaptive immune molecules, including molecules regulating immune cell migration or adhesion, in the PIA model.

In our study, we provide the first analysis of four immune molecules (IL7Rα, L-selectin, CD34, CD103) in the PIA model, to determine the effect of altered adaptive responses and cell migration on food-induced anaphylaxis. IL7Rα (CD127) is expressed on lymphoid cells and plays key roles in regulating lymphoid development, survival and proliferation [9-11]. L-selectin (CD62L) is constitutively expressed on leukocytes and involved in neutrophil extravasation [12], lymphocyte rolling and migration into lymph nodes [13] and pathology associated with T cell-mediated inflammation in a number of disease models [14,15]. CD34 is widely used as a clinical marker for the enrichment of human hematopoietic stem cells and a marker of pluripotency. However, CD34 is also expressed by a range of hematopoietic cells and vascular endothelia and promotes optimal immune cell migration (particularly for mast cells, eosinophils and dendritic cells) and the maintenance of vascular integrity [16-20]. CD103 (integrin alpha E) is expressed on subsets of dendritic cells (DCs) and lymphocytes within the gut tissues, where it acts as an E-cadherin ligand and has been proposed as a key molecule regulating oral tolerance (detailed by Scott *et al.*[21]).

Here we have performed a survey of mice deficient in these immunoreceptors to identify pathways that alter susceptibility to PIA. Our findings demonstrate that ablation of either *IL7R* or *L-sel* dramatically reduces the severity of PIA, whereas ablation of two migration-associated immune genes, *Cd34* or *Cd103,* has no effect. These findings suggest that L-selectin and IL-7R〈 play key roles in the development of adaptive immune responses to peanut antigen, while immune cell migration via CD34 or CD103-dependent mechanisms are not required. When considering effective points of intervention in PIA, our findings suggest minimal benefit in targeting late-phase immune cell migration.

## Materials and methods

### Mice

C57BL/6, CD103 (*Cd103*^*−/−*^*)* and IL-7 receptor (*IL7R*^−/−^) deficient mice were purchased from The Jackson Laboratory. *IL7R*^−/−^ mice were backcrossed onto a Ly5.1 background. L-selectin deficient (*L-sel*^−/−^) [22] mice were provided by Dr. H.J. Ziltener and CD34 deficient (*Cd34*^*−/−*^) mice [23] were provided by Dr. T.W. Mak. All animals were housed and bred in specific pathogen-free conditions at The BRC. For all experiments, eight to ten week old sex-matched mice were used and the Committee on Animal Care at UBC approved all procedures, in accordance with the requirements of the Canadian Council on Animal Care.

### Peanut-induced anaphylaxis (PIA)

PIA was induced as previously described [5]. Briefly, mice were sensitized by oral gavage with 1 mg peanut protein (Kraft Naturals peanut butter) and 10 μg cholera toxin (List Biological Laboratories) in 100μL sterile dH_2_O, weekly for 4 weeks. Control mice received PBS alone. Two weeks after the final sensitization, mice were challenged by intraperitoneal injection of 5 mg crude de-fatted peanut extract (CPE; Greer Laboratories) in 500μL PBS.

### Clinical scoring

Symptoms were evaluated using the scoring system described previously [5]. Animals were housed individually and observed for temperature decreases and development of clinical symptoms for 40 minutes post-challenge. Rectal temperatures were measured using a traceable expanded range digital thermometer (VWR) at 10-minute intervals. Clinical scores were assigned from 0–5, where 0 = no symptoms, 1 = repetitive scratching of the ear canals, 2 = decreased activity or puffiness of the eyes, 3 = periods of motionlessness for >1 minute, 4 = no response to whisker stimuli/prodding and 5 = early endpoint triggered by seizures or convulsion.

### Blood analysis (Histamine, Total IgE and IgE-mediated CPE binding)

Blood was collected via cardiac puncture from anaesthetized animals and diluted in 50ul PBS containing 2.5U of heparin. Plasma was separated by centrifugation and stored at -20C. Histamine levels were determined using an enzyme immunoassay kit (Beckman Coulter / Immunotech). Total IgE was assessed by ELISA using a murine total IgE kit (BD Pharmingen, San Diego CA). IgE-mediated CPE binding was assessed using a sandwich ELISA, similar to the protocol previously described [5]. Briefly, plates were coated with anti-mouse IgE Ab (Southern Biotech) overnight. Diluted serum samples were then incubated overnight, coated with biotinylated CPE (Greer), followed by streptavidin-alkaline phosphatase (Invitrogen) and developed with a commercial ELISA amplification system (Invitrogen). Resulting optical densities were adjusted to a standard curve of biotinylated CPE.

### Passive systemic anaphylaxis (PSA)

PSA was performed as previously described [24]. For histamine assessment, mice were sensitized by intravenous injection of 2 μg of anti-DNP IgE (Sigma-Aldrich) in 200 μl HBSS. For body temperature assessments, mice were sensitized with 60 μg of anti-DNP IgE (in-house, clone SPE-7) in 200 μl HBSS. Anaphylaxis was induced the next day by intravenous injection of 0.5-1.0 mg DNP-HSA in 200μL HBSS. Anaphylaxis severity was assessed by measuring rectal temperatures at 5-minute intervals for 60 minutes or assessment of serum histamine levels 5 minutes post-injection.

### Statistical analysis

*P* values were calculated using unpaired two-way Student’s *t* test.

## Results

### Reduced PIA pathology in *IL7R*^−/−^ and *L-sel*^−/−^ mice, but not Cd34^−/−^ or Cd103^−/−^ mice

**Figure 1:**
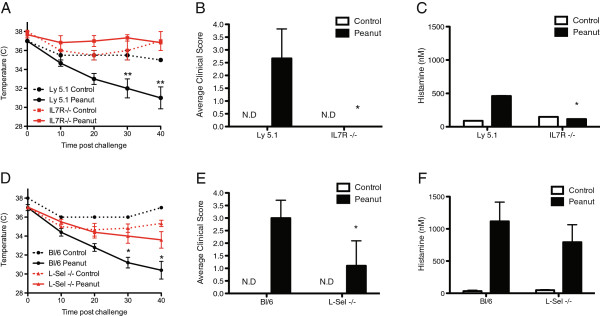
**IL-7Rα and L-selectin, are required for murine peanut-induced anaphylaxis.** Mice were initially sensitized using peanut antigen and cholera toxin via oral gavage for 4 consecutive weeks. Two weeks following the final sensitization, mice were challenged *i.p.* with crude peanut extract. Body temperature (**A**, **D**) and average observed clinical scores (**B**, **E**) monitored every 10 minutes for 40 minutes post-injection. Following the 40-minute endpoint, blood levels of histamine were assayed (**C**, **F**). Control mice were challenged with peanut immediately before monitoring. (*IL7R*^*−/−*^ and Ly5.1 *n* = 3, representative of 4 experiments; *L-Sel*^*−/−*^*n* = 8, Bl/6 *n* = 7, and control mice n = 3, representative of 5 experiments; *represents p < 0.05; **represents p < 0.01; Error bars = SEM).

Initially, we performed a survey of *IL7R*^*−/−*^, *L-sel*^*−/−*^, *Cd34*^*−/−*^ and *Cd103*^*−/−*^ mice to determine susceptibility to PIA. As previously reported, naïve mice challenged with CPE, regardless of genotype, did not exhibit any significant changes in body temperature, clinical symptoms or histamine levels when compared to control mice (Figure 1 and data not shown) [5]. To further understand of B cell and T cells in PIA [5], we assessed disease susceptibility in *IL7R*^−/−^ mice, which exhibit major defects in lymphoid development [9-11]. In wildtype (Ly5.1) mice, antigen challenge resulted in rapid decreases in body temperature (Figure 1A), observable clinical symptoms (Figure 1B) and elevated serum histamine (Figure 1C). In sharp contrast, *IL7R*^−/−^ mice were protected from disease, exhibiting limited or no decrease in body temperature (Figure 1A), no clinical symptoms (Figure 1B) and reduced histamine levels (Figure 1C).

We next assessed PIA in *L-sel*^*−/−*^ mice, as L-selectin is required for homing and migration of naïve lymphocytes and inflammatory immune cells in allergic models [13,25-27]. As such, we hypothesized L-selectin plays a role during either the sensitization stage or antigen challenge stage of disease. Following intraperitoneal challenge, *L-sel*^*−/−*^ mice exhibited a minimal temperature drop (Figure 1D), which recovered by the 40-minute endpoint, and exhibited lower average clinical scores (Figure 1E), compared to wild type controls. However, at the endpoint, *L-sel*^*−/−*^ mice exhibited elevated levels of serum histamine (Figure 1F) equivalent to wild type controls.

**Figure 2:**
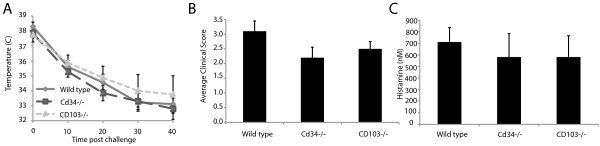
**CD103 or CD34 are not required for murine peanut-induced anaphylaxis.** Mice were sensitized with peanut antigen and cholera toxin via oral gavage for 4 consecutive weeks and were challenged *i.p.* with crude peanut extract 2 weeks following the final sensitization. Body temperature decreases (**A**) and average clinical scores (**B**) monitored every 10 minutes; observed for 40 minutes post-injection. Following the 40-minute endpoint, blood levels of histamine were assayed (**C**). (*Cd34*^*−/−*^ n = 10, *Cd103*^−/−^ n = 4, Bl/6 n = 10, representative of 2–3 experiments. *represents p < 0.05; **represents p < 0.01; Error bars = SEM).

CD34 plays a key role in mast cell migration and development of allergic asthma [16,17], so we hypothesized that *Cd34*^*−/−*^ mice would also be protected from PIA. However, following challenge, *Cd34*^*−/−*^ mice exhibited equivalent decreases in body temperature (Figure 2A), clinical scores (Figure 2B) and serum histamine levels (Figure 2C) to wildtype control mice. 

CD103 is expressed on immune cells within the gut mucosa and is a marker of DCs that maintain oral tolerance [21]. Following PIA induction, like *Cd34*^*−/−*^ mice, *Cd103*^*−/−*^ mice exhibited wildtype decreases in body temperature (Figure 2A), clinical symptoms (Figure 2B) and serum histamine levels (Figure 2C). From this initial screen, we determined that IL7R〈∀ and L-selectin are critical for PIA, but neither CD34, nor CD103, play major roles in the development of peanut-specific immunity or resulting anaphylactic responses following antigen exposure.

### Wildtype levels of total and antigen-specific IgE in *IL7R*^−/−^ and *L-sel*^−/−^ mice

**Figure 3:**
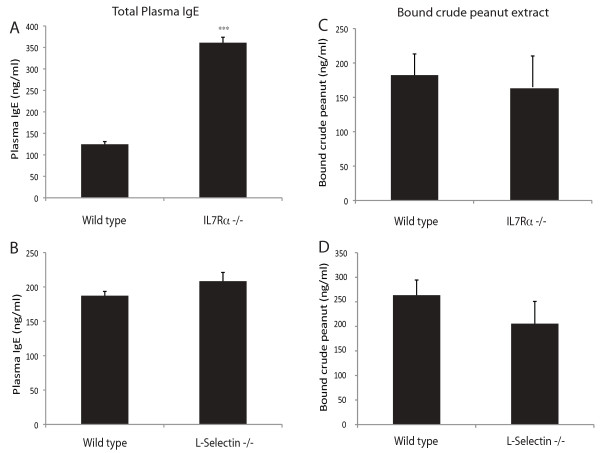
**Total and antigen-specific IgE in plasma of *IL7R***^**−/−**^** and *L-sel***^***−/−***^** mice.** Following peanut sensitization and challenge, blood levels of total IgE (**A**, **B**) and IgE-mediated CPE binding (**C**, **D**) were assayed at the 40 minute endpoint. (*IL7R*^−/−^*n* = 3 and Ly5.1 *n* = 4, representative of 3 experiments; *L-Sel*^−/−^*n* = 5 and Bl/6 *n* = 7, representative of 4 experiments; ***represents p < 0.001; Error bars = SEM).

As disease progression was impaired in *IL7R*^*−/−*^ and *L-sel*^*−/−*^ mice, we also assessed total IgE production and peanut-specific IgE binding in these animals. Surprisingly, despite decreased disease severity and histamine release, both *IL7R*^*−/−*^ and *L-sel*^*−/−*^ mice exhibited normal or increased total plasma IgE levels, compared to wildtype controls (Figure 3A,B) and normal levels of IgE-mediated CPE binding (Figure 3C,D). Thus, reduced disease severity in *IL7R*^*−/−*^ and *L-sel*^*−/−*^ mice is independent of the ability to produce peanut-specific IgE responses and may reflect lower affinity IgE production or otherwise impaired immune responses.

### L-sel^−/−^ and IL7R^−/−^ mice are fully susceptible to passive system anaphylaxis (PSA)

**Figure 4:**
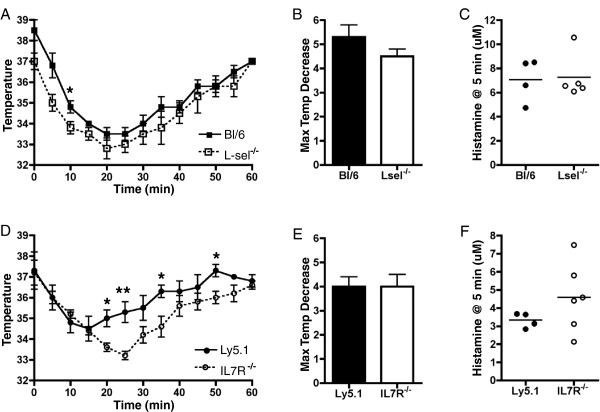
**Equivalent passive systemic anaphylaxis induction in *L-sel***^***−/−***^** and *IL7R***^***−/−***^** mice.** Mice were initially injected *i.v.* with anti-DNP IgE to simulate sensitization. The next day, mice were injected *i.v.* with DNP-HSA to induce passive systemic anaphylaxis. Body temperature measurements in *L-sel*^*−/−*^ (**A**) and *IL7R*^*−/−*^ (**D**) mice were taken at 5-minute intervals for 60 minutes post-injection. We identified maximal individual temperature decreases (**B**, **E**) and assessed serum histamine levels (**C**, **F**) in animals sacrificed 5 minutes post-injection. (n = 4-5, *represents p < 0.05; **represents p < 0.01; Error bars = SEM)

L-selectin and IL7R〈∀-deficient animals exhibited decreased susceptibility to PIA, which could result from impaired immune sensitization or impaired anaphylactic responses. To test the latter possibility, we assessed the susceptibility of *L-sel*^−/−^ and *IL7R*^−/−^ mice to a PSA model. Mice were loaded with anti-DNP IgE and challenged with DNP-HSA. After challenge, both *L-sel*^*−/−*^ and *IL7R*^*−/−*^ exhibited marked decreases in body temperature, which recovered to initial body temperatures, similar to their respective wildtype Bl/6 and Ly5.1 controls (Figure 4A,D) and individual maximal temperature decreases were indistinguishable across genotypes (Figure 4B,E). Further, assessment of serum histamine levels 5 minutes post-challenge revealed equivalent levels of histamine release in all animals tested (Figure 4C,F). These findings demonstrate that while *L-sel*^*−/−*^ and *IL7R*〈 ∀ mice are protected from PIA, both strains are capable of mounting a robust systemic anaphylactic response when loaded with equivalent levels of antigen-specific IgE.

## Discussion

Peanut-induced anaphylaxis is a severe medical condition, with major effects on individual patient health and social policy. Despite this, we lack a basic understanding of unique underlying mechanisms of the disease. Recent studies using a mouse model of PIA have highlighted the role of B cells in peanut allergy development or focussed on potential therapeutic interventions [5-7]. However, the importance of other specific immune molecules and immune processes underlying this disease are not well understood. In the current study, we used a series of knockout mice to survey the importance of cytokine receptors and adhesion/trafficking molecules in susceptibility to food allergy with the goal of identifying novel pathways as points of therapeutic intervention.

We were surprised to find that ablation of CD34 had no effect on PIA, since this molecules has previously been shown to play critical roles in a variety of immune cell mediated disease models [16,18,28,29]. One possible explanation is that after priming is complete, cell trafficking no longer plays a role in the effector phase of PIA due to the short timeline of disease (~1 hour). Although CD34 facilitates the migration of several hematopoietic effector lineages (mast cells, eosinophils and DCs) and *Cd34*^−/−^ mice are protected in models of asthma, ulcerative colitis and hypersensitivity pneumonitis, this likely reflects a *delay, but not a block,* in the ability of CD34^+^ effector cells to migrate [16,18,28,29]. Thus, given sufficient time for priming, it is likely that these mice “catch up” to their wildtype counterparts and are equally susceptible to the acute phase of an anaphylactic response.

CD103 is an E-cadherin ligand proposed to specify tissue localization of CD103^+^ DCs and mucosal T cells within the gut [30-34]. CD103^+^ DCs promote both T_reg_ development and T_eff_ cell homing [30,32], and play a key role in maintaining oral tolerance and gut homeostasis. Notably, while CD103 is a marker of T_reg_ subsets, recent work has demonstrated that CD103 does not play an essential role in T_reg_ mediated functions in the gut. Mullaly *et al.*, demonstrated that CD103 was not required for immune responses during helminth infection, and that mice which lack CD103 have normal levels of T_reg_ in the mesenteric lymphnodes or lamina propria [35]. CD103 is also a marker of pro-regulatory DCs, however very little work has focused on the functional role of CD103 on these cells. Therefore we and others [21], hypothesized that *Cd103* ablation would exacerbate disease, if pro-regulatory DCs modulate disease severity. Our findings demonstrate, however, that loss of CD103 has no effect on PIA disease severity and, thus, we propose that CD103 is a valuable marker of DC and T_reg_ subsets in the gut, but does not play an essential role in the development or maintenance of oral tolerance.

Our findings demonstrate that disease severity is reduced when adaptive immune responses are impaired. This is particularly evident in *IL7R*^*−/−*^ mice, which exhibit severe defects in lymphoid development and survival, resulting in lymphopenia, low thymic cellularity and impaired antibody production [9-11]. Surprisingly, despite severe defects, *IL7R*^*−/−*^ mice produce normal serum Ig levels [36] and in the PIA model had wildtype (or elevated) levels of both total IgE and antigen-specific IgE. Nevertheless, *IL7R*^*−/−*^ mice exhibited reduced circulating histamine levels following peanut challenge. This apparent discrepancy may reflect a severely limited antibody repertoire in *IL7R*^*−/−*^ mice [37]. Without effective IL-7R signalling, distal regions of the immunoglobulin heavy chain loci become inaccessible, resulting in limited B cell repertoires and, likely, lower affinity antibody production [37]. *In vivo,* lower affinity antibodies likely fail to induce a robust histamine release (despite normal IgE levels), resulting in an absence of clinical symptoms in the PIA model. This finding is consistent with the known role of adaptive immunity in allergic responses, and the importance of B cells reported in this model [5].

The degree of protection from PIA was more subtle in *L-sel*^*−/−*^ mice, which exhibit an attenuated response. L-selectin is involved in adaptive immunity both in naïve T cell homing and the migration of mature Ag-specific T cells during inflammation [12-14,26,27]. *L-sel*^*−/−*^ mice mount a normal antibody response [14], but are protected in a number of acute inflammatory and T-cell mediated models of delayed-type hypersensitivity reaction and experimental allergic encephalomyelitis [14,15,25-27]. Intriguingly, despite reduced disease pathology in *L-sel*^*−/−*^ mice, no difference in blood histamine or IgE levels was observed following PIA-induction, suggesting that they may represent an asymptomatic sensitization to peanut allergen [38]. Our findings also suggest that protection in *L-sel*^*−/−*^ mice is via a histamine-independent mechanism, (most likely regulating T cell or neutrophil recruitment and migration). Intriguingly, despite the reduced susceptibility to PIA, both *IL7R*^−/−^ and *L-sel*^−/−^ mice exhibited normal susceptibility to passive systemic anaphylaxis.

In summary, we have shown that targeting L-selectin or IL7R〈∀ function is sufficient to reduce PIA responses, while loss of CD34 or CD103 has no effect on disease severity. More broadly, these findings suggest that interventions targeting initial immune sensitization are more likely to meet with therapeutic success, by suppressing effective antigen-specific antibody production and inhibiting late-phase anaphylaxis/mast cell responses. Conversely, therapies inhibiting immune cell migration following antigen challenge are unlikely to have significant benefits, particularly considering the rapid kinetics of peanut-induced anaphylaxis.

## Conflict of interest

No conflict of interest is declared by any authors of this manuscript.

## Authors’ contributions

SM and JB designed and performed all experiments and wrote the manuscript. EDB coordinated reagent procurement and initiation of the project, participated in experiment harvests and edited the manuscript. MJG, MCT, and ZJ performed selected assays and edited the manuscript. JSM and KMM supervised trainees, edited the manuscript, and provided reagents. All authors read and approved the final manuscript.
